# Supplementary material for: Construction and Evaluation of a Novel Organic Anion Transporter 1/3 CRISPR/Cas9 Double-Knockout Rat Model
Source: Pharmaceutics. 2022 Oct 27;14(11):2307. doi: 10.3390/pharmaceutics14112307 (PMC9697873; doi:10.3390/pharmaceutics14112307)
Supplement: Supplementary file 1 [file pharmaceutics-14-02307-s001.zip › pharmaceutics-1940245-supplementary.pdf]

Table S1. Mass spectrometry parameters of uremic toxins.

Name	Precursor ion(m/z)	Product ion(m/z)	Fragmentor /V	Collision energy/V	Polarity
N6-(carboxymethyl)-l-lysine	205.2	84.1	90	18	positive
Creatinine	114	44.2	70	16	positive
S-adenosyl-L-homocysteine	385	136	100	14	positive
N-acetyl-L-arginine	217.1	70	110	30	positive
D-neopterin	254	205.9	90	12	positive
3-(3,4-dihydroxyphenyl)-L-alanine	198	151.9	80	8	positive
DL-homocysteine	136	89.8	70	5	positive
Uridine	267	134.8	90	8	positive
1-methyl-inosine	305	173	190	10	positive
Uric acid	206.9	81.7	160	34	positive
Orotic acid	155	111.2	60	16	positive
1-methyl-5-carboxylamide-2-pyridone	153	108	120	20	positive
N2,N2-dimethylguanosine	312.1	180	90	8	positive
D-kynurenine	209.1	145.9	80	8	positive
N-acetylcytidine	308	176	110	8	positive
3-Indolyl-β-D-glucopyranoside	318	256.1	120	18	positive
Phenyl-β-D-glucuronide	269.6	93.2	90	30	negative
P-cresol glucuronide	283.7	107.2	90	30	negative
Phenylacetyl-L-glutamine	263.6	145.4	100	4	negative
3-Indoxyl sulfate potassium salt	212.4	80	90	14	negative
Hippuric acid	178.1	134	65	8	negative
4-Ethylphenyl sulfate	201.5	121.2	90	4	negative
P-cresol sulfate	186.99	106.82	100	30	negative
N-(cinnamoyl) glycine	203.9	103.1	80	6	negative
Indole-3-acetic acid	174.4	130.3	70	4	negative
CMPF	239.5	195.2	90	3	negative

CMPF, 3-Carboxy-4-methyl-5-propyl-2-furanpropionic acid;

Table S2. Primers information of Q-PCR.

No.	Gene name	Primer sequence (5′ – 3′)
1	*Slc22a1*/Oct1	F: TGGCCGTAAGCTCTGTCTCT
		R: TCAAGGTATAGCCGGACACC
2	*Slc22a2*/Oct2	F: GCAAGCAGACCGTCCGCTAAG
		R: CAGACCGTGCAAGCTACAGCTC
3	*Slc22a6*/Oat1	F: GGCACCTTGATTGGCTATGT
		R: CCACAGCATGGAGAGACAGA
4	*Slc22a7*/Oat2	F: CGCTCAGAATTCTCCTCCAC
		R: ACATCCAGCCACTCCAACTC
5	*Slc22a8*/Oat3	F: GCATCCACCTCCAGTCCAAC
		R: AGGGCCAGCAAGGTCACATG
6	*Slc22a11*/Oat4	F: GAATTCAACAGATGTCC

		R: TGGCTGGAATGGTGATTATACC
7	*Slc22a12*/Urat1	F: TCCCCACTGTGATCAGGATGA
		R: AATGCAGCTAGGCCGCTTAAC
8	*Slc47a1*/Mate1	F: CTCTTCATCAACACCGAGCA
		R: ACCCATCACCCCAAGATGTA
9	*Slco4c1*/Oatp4c1	F:GCAAGGTATTGTAGTAAATGGCCTAGT
		R: AGACAACACGCAAAAGGAGATG
10	*Abcc2*/Mrp2	F: AAACGTTCACGGGCACATC
		R: CAGGACTGCTGAGGGACATAGG
11	*Abcc4*/Mrp4	F: GGACACTGAACTAGCAGAATC
		R: TGTATTAACTCGTCAGTTCTCG
12	*Abcb1a*/Mdr1a	F: GCAGGTTGGCTGGACAGATT
		R: GGAGCGCAATTCCATGGATA
13	*Abcb1b*/Mdr1b	F: CTGCTATCATCCACGGAACC
		R: GCTGACGGTCTGTGTACTGTTG
14	*Abcg2*/Bcrp	F: TTGGACTCAAGCACAGCAAAT
		R: ATGGAATACCGAGGCTGGTGA
15	β-Actin	F: GTCGTACCACTGGCATTGTG
		R: AGGAAGGAAGGCTGGAAGAG

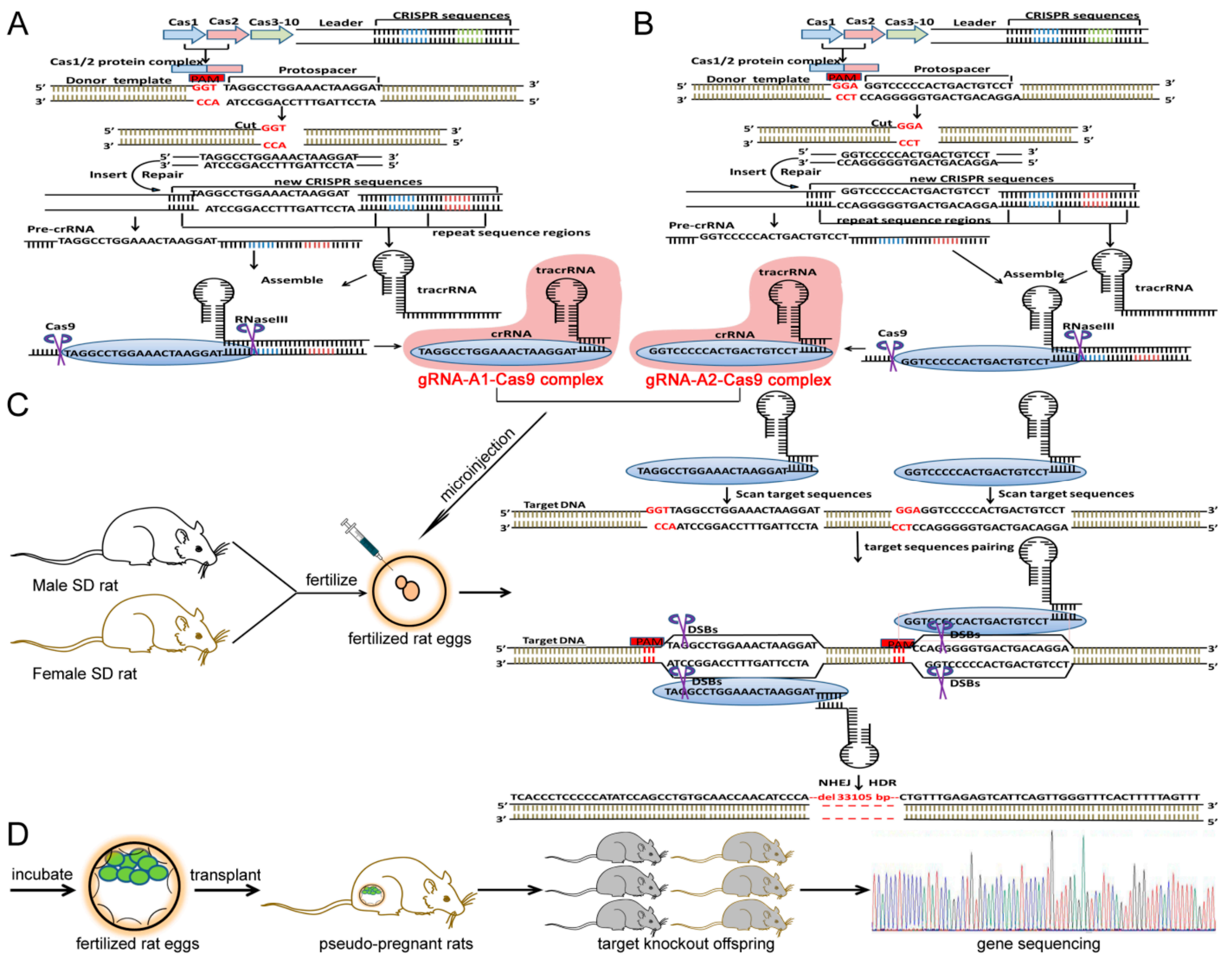

Figure S1. Schematic diagram of the CRISPR/Cas9-mediated *Slc22a6*/*Slc22a8* knock-out. (**A**) Synthesis of *gRNA*-A1

and *gRNA*-A1-Cas9 complex. (**B**) Synthesis of *gRNA*-A2 and *gRNA-A2*-Cas9 complex. (**C**) The *gRNA* recognizes the target gene sequence, the Cas9 endonuclease efficiently cleaves the target gene sequence, non-homologous end joining (NHEJ) and homology-directed repair (HDR). (**D**) Generation of targeted knockout offspring. PAM, protospacer adjacent motif; pre-*crRNA*, pre-CRIPSR-derived *RNA*; *tracrRNA*, trans-acting *RNA*; *gRNA*, guide *RNA*.

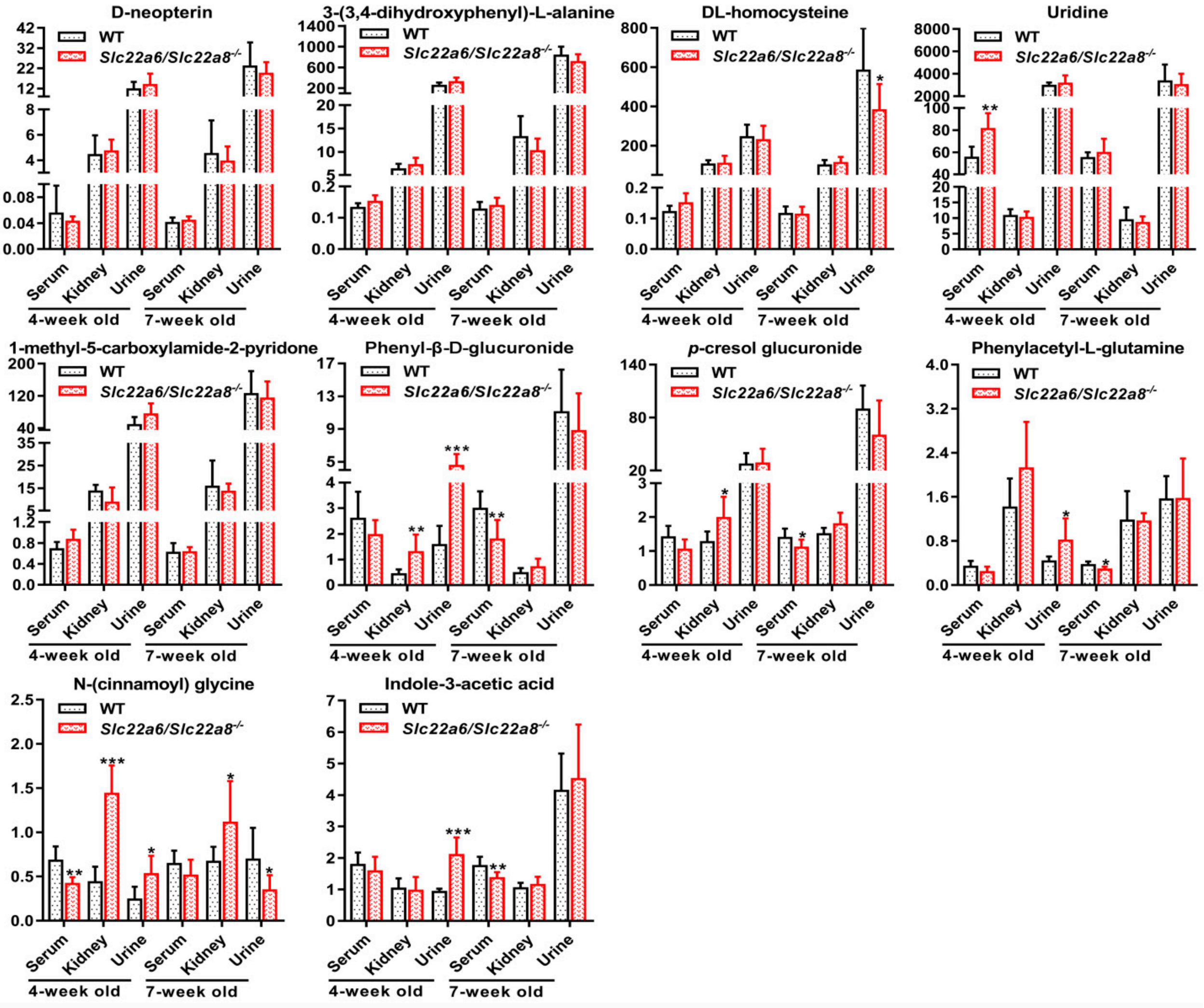

Figure S2. Effects of *Slc22a6*/*Slc22a8* double-knockout on the endogenous UTs (D-neopterin, DL-homocysteine, 3-(3,4-dihydroxyphenyl)-L-alanine, uridine, 1-methyl-5-carboxylamide-2-pyridone, p-cresol glucuronide, phenyl-β-D-glucuronide, phenylacetyl-L-glutamine, N-(cinnamoyl) glycine, indole-3-acetic acid) in 4W and 7W WT and KO rats. Plasma, urine concentration and kidney uptake ratio of UTs in 4W and 7W WT and KO rats. The unit of the plasma and urine concentration of UTs is ug/ml and the renal uptake ratio is ml/g. Data are presented as mean ± SD ($n = 7$). *, $p < 0.05$; **, $p < 0.01$; ***, $p < 0.001$ compared with WT rats.

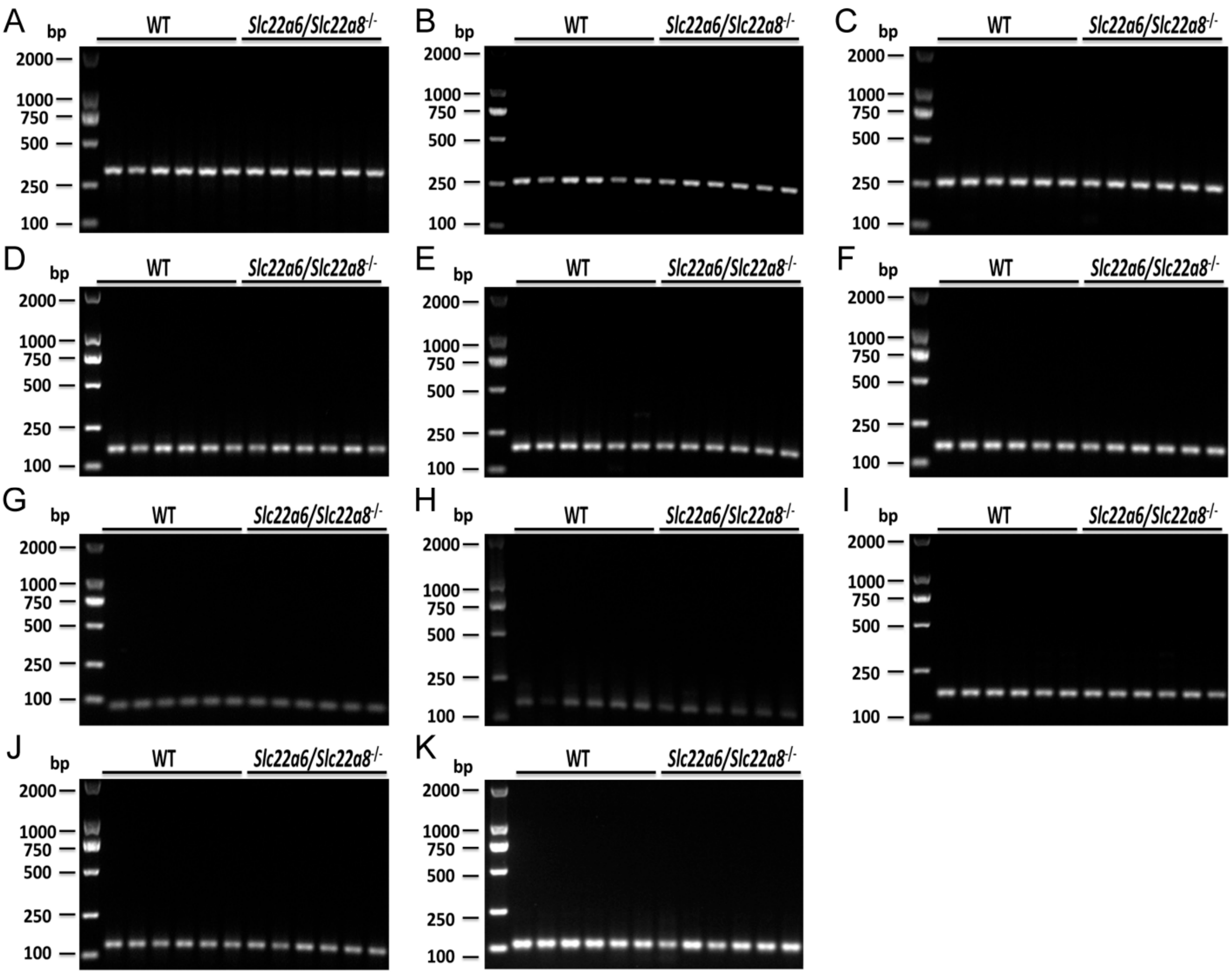

Figure S3. Validation of the specificities of the primers used in the Q-PCR. The amplified real-time quantitative PCR products were electrophoresed on a 1.5% agarose gel and imaged by an automatic chemiluminescence image analysis system (*n* = 6). (**A**) *Slc22a7*. (**B**) *Slc22a11*. (**C**) *Slc47a1*. (**D**) *Slc22a1*. (**E**) *Slc22a2*. (**F**) *Abcc4*. (**G**) *Mdr1a*. (**H**) *Mdr1b*. (**I**) *Slc22a12*. (**J**) *Slco4c1*. (**K**) *Abcg2*.

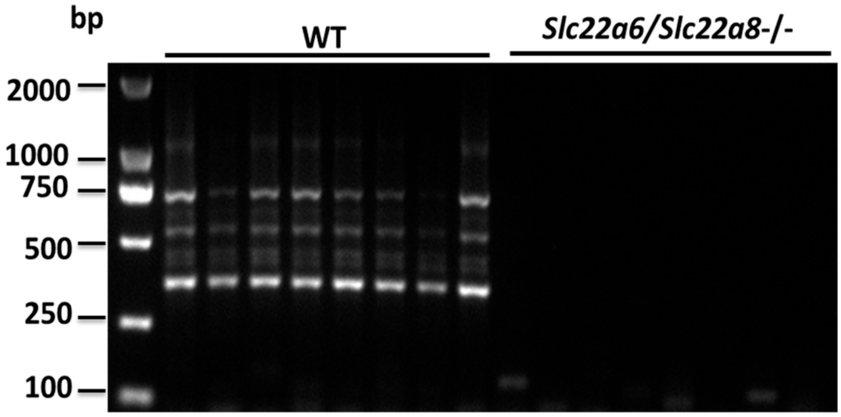

Figure S4. Agarose gel electrophoresis of the Q-PCR amplification products of *Slc22a8* primers (*n* = 8).
